# Life-sustaining treatment decisions in pediatric intensive care: an Italian survey on ethical concerns

**DOI:** 10.1186/s13052-021-01054-z

**Published:** 2021-07-07

**Authors:** Franco A. Carnevale, Alberto Giannini, Amabile Bonaldi, Elena Bravi, Costanza Cecchi, Andrea Pettenazzo, Angela Amigoni, Silvia Maria Modesta Pulitanò, Chiara Tosin, Paolo Biban

**Affiliations:** 1 McGill University/Montreal Children’s Hospital, Montreal, 680 Sherbrooke St. West, Suite 1836, Montréal, Quebec H3A 2M7 Canada; 2 S.C. Anestesia e Rianimazione Pediatrica, Ospedale dei Bambini, ASST Spedali Civili di Brescia, Brescia, Italy; 3 Department of Neonatal and Pediatric Critical Care, Verona University Hospital, Verona, Italy; 4 U.O. Psicologia, Azienda Provinciale Servizi Sanitari, Trento, Italy; 5 Pediatric Intensive Care Unit, Meyer Children’s Hospital, Florence, Italy; 6 Pediatric Intensive Care Unit, Department of Woman’s and Child’s Health, University Hospital of Padova, Padua, Italy; 7 Pediatric Intensive Care Unit Trauma Center Pediatric, F. Policlinico A. Gemelli IRCCS, Università Cattolica Sacro Cuore, Rome, Italy

**Keywords:** Ethics, Critical care, Intensive care, Italy, Life-sustaining treatment decisions, Pediatric

## Abstract

**Objectives:**

To investigate how life-sustaining treatment (LST) decisions are made and identify problematic ethical concerns confronted by physicians and nurses in pediatric intensive care within Italy.

**Methods:**

An 88-question online survey was created, based on a previous qualitative study conducted by this team. The survey was designed to identify how LST decisions were managed; contrasting actual practices with what participants think practices should be. Replies from physicians and nurses were compared, to identify potential inter-professional ethical tensions. The study also identified participants’ principal ethical concerns. Moreover, open-ended questions elicited qualitative perspectives on participants’ views. The survey was pilot-tested and refined before initiation of the study.

**Results:**

31 physicians and 65 nurses participated in the study. Participants were recruited from pediatric intensive care units across five Italian cities; i.e., Florence, Milan, Padua, Rome, Verona. Statistically significant differences were identified for (a) virtually all questions contrasting actual practices with what participants think practices should be and (b) 14 questions contrasting physician replies with those of nurses. Physicians and nurses identified the absence of legislative standards for LST withdrawal as a highly problematic ethical concern. Physicians also identified bearing responsibility for LST decisions as a major concern. Qualitative descriptions further demonstrated that these Italian pediatric intensive care clinicians encounter significantly distressing ethical problems in their practice.

**Conclusions:**

The results of this study highlight a need for the development of (a) strategies for improving team processes regarding LST decisions, so they can be better aligned with how clinicians think decisions should be made, and (b) Italian LST decision-making standards that can help ensure optimal ethical practices.

## Background

### NB: this study was conducted before the COVID-19 pandemic. Therefore, pandemic-related ethical concerns are not reflected in this investigation

It is widely recognized that critical illness in childhood commonly requires complex health care that frequently gives rise to challenging ethical concerns [1–4]. A leading ethical concern relates to the use or withdrawal of life-sustaining treatments (LSTs), such as assisted ventilation (invasive or non-invasive), chest compressions, inotropic support of circulatory function, renal replacement therapies, parenteral or enteral nutrition or hydration, extra-corporeal membrane oxygenation, and selected surgical interventions.

In many Western countries, ethical standards relating to treatment decisions for children are based on the child’s ‘best interests’ [5, 6]. Best interests is commonly defined as the treatment option that offers the greatest proportion of benefit in relation to burden. Generally, these standards recognize that any LST can be withheld or withdrawn depending on the balance of benefits and burdens for the child [1, 6, 7]. However, a child’s best interests are frequently difficult to determine because it can be unclear which benefits and burdens should carry the greatest weight. Moreover, it sometimes unclear what decisional authority and responsibility should be borne by different stakeholders when making LST decisions with regard for critically ill children; e.g., parents, physicians, nurses and other health care providers (HCPs), as well as child-patients themselves. A statement published by the Italian Society of Neonatal and Pediatric Anesthesia and Intensive Care states that the physician in charge of the patient’s care and the unit head bear the main responsibility for the final decision, although the participation of other staff and the parents should be sought [2].

This investigation followed a previous qualitative study by the authors in Italy [8], wherein focus groups with 16 physicians and 26 nurses as well as individual interviews with 9 parents were conducted. Findings uncovered the ‘private worlds’ of pediatric intensive care unit (PICU) physicians, nurses and parents. As they struggled through complex ethical dilemmas, they all suffered tremendously and privately. Physicians struggled with the weight of responsibility and solitude in making LST decisions. Nurses struggled with feelings of exclusion from decisions regarding the patients and families that they cared for. Physicians and nurses were distressed by legal barriers to LST withdrawal. Parents struggled with their dependence on physicians and nurses to provide care for their child, striving to understand what was happening to their child.

Aside from this 2011 study, very little empirical research has examined ethical concerns in Italian pediatric intensive care. Our previous study demonstrated that there are significant and under-examined ethical concerns in the PICU that require further investigation.

The objective of this study was to investigate how ethical concerns are managed in Italian pediatric intensive care. Specifically, we examined how LST decisions are made and sought to identify the most problematic ethical concerns confronted by Italian physicians and nurses in the PICU.

## Methods

### Questionnaire development

An online survey questionnaire was developed by the research team. The questionnaire was designed with the survey software LimeSurvey, on a secure password-protected server. The LimeSurvey online survey tool was hosted on a McGill University (Montreal, Canada) server and maintained by the Service Centre Tools Implementation group.

Themes that were identified in our initial qualitative study were used to develop the questionnaire. The aim was to develop a questionnaire that would: (a) be succinct and not require more than 10 min to complete; (b) collect some general descriptive information about participants; (c) document participant’s perceptions about *actual* practices regarding LST decision-making and their thoughts about how these *should be* made; and (d) identify ethical concerns that participants consider most problematic.

The questionnaire was designed to collect data for two comparative analyses: (a) responses between physicians and nurses, as well as (b) reported ‘actual’ and ‘should be’ practices for all participants.

Upon completion of the first version of the questionnaire, a first pilot testing of the online questionnaire was conducted with three Italian PICU nurses, to assess the clarity, time-requirement, and technical functionality of the online survey. The survey was further adapted and a second pilot testing was conducted with 2 physicians and 2 nurses working within an Italian PICU. The second pilot test examined the following questions (English translation of pilot test conducted in Italian): (a) How long did it take to complete the questionnaire?; (b) In your opinion, is this time excessive or adequate?; (c) Did you find it difficult to answer any questions? If so, which ones and for what reason? (response options: difficult to understand; difficult to relate to my reality; too complex); (d) What would you change in the questionnaire structure? (i.e., sections to be deleted and/or added and/or modified); and (e) Do you want to add your own comments on the questionnaire?

The final version of the online questionnaire consisted of 88 items regarding LST decisions, as well as questions on participants’ demographic background.

### Sampling and participant recruitment

It was recognized that social ethical viewpoints and underlying moral values could vary across different cities and regions in Italy. It was also believed that ethical views on various clinical practices could vary across settings. It was important to ensure that sampling for this study would include multiple settings. Therefore, a total of five PICUs from five cities were recruited to participate; i.e., Florence, Milan, Padua, Rome, Verona (Table 1). Although PICUs from southern Italy were also invited to collaborate, none agreed to participate during the study’s recruitment period despite repeated requests.

**Table 1:** Participating Italian PICUs

City	Hospital Center	Participants
Padua	Azienda Ospedaliera di Padova	19
Florence	Azienda universitaria ospedaliera Anna Meyer	24
Verona	Azienda Ospedaliera Universitaria di borgo Trento Verona/Ospedale Civile Maggiore Verona/Azienda ospedaliera	27
Rome	Fondazione Policlinico “A. Gemelli”	16
Milan	Fondazione IRCCS Ca′ Granda Ospedale Maggiore Policlinico	12

Moreover, participating PICUs were solicited in a manner that could ensure a mix of PICUs with *anesthetist-intensivists* as well as *pediatrician-intensivists*. These two different training backgrounds were believed to be potentially associated with different clinical practice approaches; although this had not been systematically documented.

The diverse mix of different cities and physician training backgrounds were used solely to ensure that the participating sample was inclusive of these PICU diversities. These factors were not examined for statistical differences, as this would require a significantly larger sample size and more complex analyses.

Two lead physicians on this research team are leaders within the Italian PICU community; each brought a different training background to the study (Biban: pediatrician-intensivist; Giannini: anesthetist-intensivist). One of the nurses on the research team (Bonaldi) is an active member of the Italian PICU nursing community. The two lead physicians (Biban; Giannini) prepared a list of PICUs in Italy that met the sampling requirements described above, striving to recruit a minimum of 50 physicians and 50 nurses (i.e.: based on the design of the survey scales, t-test analyses were planned to examine statistical significance. For a d = 0.5, where we considered a difference of 1.0 between physicians and nurses as moderately significant on a 5-point scale and a Power of 0.8; a minimum total sample size of 100 was required; 50 physicians and 50 nurses). The medical director for each identified PICU was contacted, to solicit the PICU’s participation in the study. For each PICU where the medical director agreed to participate, the lead Italian nurse on the team (Bonaldi) contacted the nurse manager in that PICU to solicit the participation of the nurses in that PICU.

The goal was that the medical director and the nurse manager in each participating PICU would promote the study in their PICU and help recruit physicians and nurses to participate in the study. The medical directors and nurse managers were sent a short announcement that they could distribute among physicians and nurses. The announcement provided a brief description of the study and indicated a direct link to the online questionnaire. These announcements were circulated by email and/or by hard copy, depending on the preferences of each PICU.

Following repeated recruitment measures over the course of several months, recruitment was terminated, as the investigators were concerned that an overly prolonged data collection period could result in ‘data contamination’ of potential practice changes over time. A total of 31 physicians and 65 nurses were recruited.

### Statistical analysis

Given that (a) the required sample sizes for t-test analyses were not attained and (b) all data were rated on a 5-point scale (ranging from 1 = strongly disagree to 5 = strongly agree) and were not normally distributed, data were analyzed with nonparametric robust statistical methods by a specialized statistician who ran a series of between-person and within-person comparisons [9]. The statistician was naive to the specific hypothesis for each comparison. All comparisons conducted were based on the masked-coded variables provided by the investigators. All robust tests were conducted using the ‘WRS’ or the ‘WRS2’ packages in R version 3.6. For each series of comparisons, a sample R code for the first comparison is provided.

Two statistical comparisons were conducted. The first involved a series of analyses comparing physician with nurse responses for all 88 questions in the survey. For this set of independent mean comparisons, Yuen’s modified t-test [10] for independent trimmed means with 5000 bootstrap was used [9–11]. In order to adjust for multiple comparisons, the Benjamin-Hochberg procedure was used [12, 13]. The original *p*-values are reported in the Appendix.

A second series of analyses were conducted to examine 27 pairs of questions in the survey for nurses and physicians separately. For this set of dependent mean comparisons, a procedure using 20% trimmed mean with a 5000 percentile bootstrap was used [14].

### Ethical considerations

The online questionnaire indicated that completion of the survey would represent the participants’ consent for their replies to be used for the study. No additional consent procedure was required.

The online questionnaire was designed in a manner that ensured the personal identity of each participant was not identifiable. Moreover, the online survey data was stored on a secure password-protected university-based server at McGill University, in Montreal (Canada), where one of the researchers was located.

The study received research ethics approval from the Ethics Committee for Clinical Experimentation for the Province of Verona and Rovigo, Azienda Ospedaliera Universitaria Integrata Verona (i.e., approval code number: 795CESC).

## Results

Table 2 outlines descriptive data regarding respondents who participated in the study, which included 31 physicians and 65 nurses practising in 5 different PICUs in different regions of Italy.

**Table 2:** Participant information

Profession	Number
Physicians	31
Nurses	65
Unspecified	2
**Gender**	**Number**
Female	70
Male	26
**Age (years)**	**Number**
20–29	16
30–39	33
40–49	31
50–59	14
60 or over	3
Unspecified	1
**Time since completion of training (years)**	**Number**
< 2	4
2–4	13
5–9	17
10–14	30
15–19	8
20–24	9
> 25	17
**Time working in this PICU (years)**	**Number**
< 2	20
2–4	9
5–9	18
10–14	27
15–19	13
20–24	8
> 25	3
**Religious orientation (self-described)**	**Number**
Religious *Includes 70 Roman Catholic	72*
Non-religious (e.g., atheist, agnostic)	26

Table 3 indicates survey questions where there was a statistically significant difference in responses between nurses and physicians. These differences were noted in 14 items, out of the total 88 items on the survey questionnaire. These differences related to:

**Table 3:** Analysis of survey data on LST decisions in Italian Pediatric Intensive Care

Question code	Survey Question (translated from actual survey in Italian) ‘*’ indicates statistical significance between nurses and physicians	Mean (RN)	Mean (MD)	Notes: Description of identified statistical differences between nurses and physicians
**How are LST decisions routinely made? Please answer the following questions by referring to the approach usually used in your ward**				
**No 12: Discussion (discussion refers to the exchange of information and/or seeking the opinions of others)**				
NO12 – AS1	The decision is discussed first with the parents [Scale 1]	3.692308	3.419355	
NO12 – AS2	The decision is discussed first with the parents [Scale 2]	3.815385	3.774194	
NO12 – BS1	The decision is discussed first with other physicians in the PICU team [Scale 1]	3.830769	4.225806	
*NO12 – BS2	The decision is discussed first with other physicians in the PICU team [Scale 2]	3.984615	4.387097	Nurses ranked this item lower than physicians.
NO12 – CS1	The decision is discussed first with the nurses [Scale 1]	3.0	3.354839	
NO12 – CS2	The decision is discussed first with the nurses [Scale 2]	3.184615	3.516129	
**No 13: Responsibility for the decision**				
NO13 – AS1	The responsibility for the decision is entrusted to the individual physician [Scale 1]	2.0	2.096774	
NO13 – AS2	The responsibility for the decision is entrusted to the individual physician [Scale 2]	1.923077	1.967742	
NO13 – BS1	The responsibility for the decision is shared with other physicians in the PICU team [Scale 1]	3.892308	4.096774	
*NO13 –BS2	The responsibility for the decision is shared with other physicians in the PICU team [Scale 2]	4.015385	4.290323	Nurses ranked this item lower than physicians.
NO13 – CS1	The responsibility for the decision is shared with the parents [Scale 1]	3.707692	3.387097	
NO13 – CS2	The responsibility for the decision is shared with the parents [Scale 2]	3.8	3.354839	
NO13 – DS1	The responsibility for the decision is shared with the nurses [Scale 1]	3.153846	3.290323	
NO13 – DS2	The responsibility for the decision is shared with the nurses [Scale 2]	3.230769	3.387097	
**No 14: Other aspects**				
*NO14 – A	In our PICU, it is permissible to not initiate LSTs	3.122449	3.138298	Nurses ranked this item lower than physicians.
NO14 – B	In our PICU, it is permissible to withdraw LSTs	3.357143	3.382979	
NO14 – C	Parents are always informed of the LSTs decision	3.918367	3.904255	
NO14 – D	When LSTs are withheld in a patient, this decision is documented in the patient record	3.663265	3.680851	
NO14 – E	When LSTs are discussed, an ethics consultation is requested	2.938776	2.914894	
**No 15: Follow-up**				
NO15 – A	After a decision regarding LSTs has been made, a follow-up meeting with the parents is planned	3.357143	3.329787	
NO15 – B	After a decision regarding LSTs has been made, a follow-up meeting with staff is planned	3.102041	3.053191	
**How SHOULD LST decisions be made?**				
**No 16: Discussion (discussion refers to the exchange of information and / or seeking the opinions of others)**				
*NO16 –BS1	The decision should be discussed first with other physicians in the PICU team [Scale 1]	4.430769	4.806452	Nurses ranked this item lower than physicians.
*NO16 –BS2	The decision should be discussed first with other physicians in the PICU team [Scale 2]	4.584615	4.967742	Nurses ranked this item lower than physicians.
NO16 –AS1	The decision should be discussed first with the parents [Scale 1]	4.169231	4.193548	
NO16 –AS2	The decision should be discussed first with the parents [Scale 2]	4.307692	4.322581	
NO16 –CS1	The decision should be discussed first with the nurses [Scale 1]	4.292308	4.419355	
NO16 –CS2	The decision should be discussed first with the nurses [Scale 2]	4.338462	4.548387	
**No 17: Responsibility for the decision**				
NO17 - AS1	The responsibility for the decision should be entrusted to the individual physician [Scale 1]	1.369231	1.354839	
NO17 – AS2	The responsibility for the decision should be entrusted to the individual physician [Scale 2]	1.384615	1.16129	
NO17 - BS1	The responsibility for the decision should be shared with other physicians in the PICU team [Scale 1]	4.492308	4.741935	
*NO17 – BS2	The responsibility for the decision should be shared with other physicians in the PICU team [Scale 2]	4.553846	4.903226	Nurses ranked this item lower than physicians.
NO17 – CS1	Responsibility for the decision should be shared with parents [Scale 1]	4.138462	3.741935	
NO17 – CS2	Responsibility for the decision should be shared with parents [Scale 2]	4.153846	3.774194	
NO17 – DS1	Responsibility for decision should be shared with nurses [Scale 1]	4.384615	4.290323	
NO17 – DS2	Responsibility for decision should be shared with nurses [Scale 2]	4.415385	4.419355	
**No 18: Other aspects**				
NO18 - A	In our PICU, it should be permissible to not initiate LSTs	4.071429	4.053191	
NO18 – B	In our PICU, it should be permissible to withdraw LSTs	4.22449	4.202128	
NO18 – C	Parents should always be informed of the decision	4.306122	4.297872	
NO18 – D	When LSTs are withheld in a patient, this decision should be documented in the patient record	4.285714	4.265957	
NO18 – E	When LSTs are discussed, an ethics consultation should be sought	4.030612	4.010638	
**No 19: Follow-up**				
NO19-A	After a decision regarding LSTs has been made, there should be a follow-up meeting with the parents	4.234694	4.223404	
NO19-B	After a decision regarding LSTs has been made, there should be a follow-up meeting with staff	4.326531	4.308511	
**No 20: Decision-making criteria** **Which criteria are used in your PICU to make these kinds of LST decisions?**				
NO20 – A	Full LSTs are provided for all patients at all times.	3.530612	3.553191	
NO20 – B	LSTs are not initiated and/or not augmented if the patient has a severe neurological injury	2.693878	2.670213	
NO20 – C	LSTs are withdrawn if the patient has a severe neurological injury	2.846939	2.829787	
NO20 – D	LSTs are not initiated and/or not augmented if the patient does not respond to treatment	2.581633	2.574468	
NO20 – E	LSTs are withdrawn if the patient does not respond to treatment	2.714286	2.712766	
NO20 – F	LSTs are not initiated and/or not augmented if it is understood that the patient will not survive the treatment	2.94898	2.925532	
*NO20 – G	LSTs are withdrawn if it is understood that the patient will not survive the treatment	3.040816	3.021277	Nurses ranked this item higher than physicians.
*NO20 – H	LSTs are not initiated and/or not augmented if the treatment would only contribute to prolonging the patient’s suffering	3.020408	3.010638	Nurses ranked this item higher than physicians.
*NO20 – I	LSTs are withheld if the treatment would only contribute to prolonging the patient’s suffering	3.081633	3.074468	Nurses ranked this item higher than physicians.
NO20 – J	LSTs are not initiated and/or not augmented if the treatment does not ensure the minimum requirements for a dignified life (for example: at least a partial relational life and autonomy, absence of uncontrolled pain)	2.673469	2.702128	
NO20 – K	LSTs are withdrawn if the treatment does not ensure the minimum requirements for a dignified life (for example: at least a partial relational life and autonomy, absence of uncontrolled pain)	2.806122	2.787234	
NO20 – L	LSTs are not initiated and/or not augmented if parents ask for LSTs to be stopped	3.081633	3.106383	
NO20 – M	LSTs are withdrawn if parents ask for LSTs to be stopped	3.040816	3.053191	
**No 22: In your opinion, what criteria do you think SHOULD be used?**				
NO22 – A	Full LSTs should be provided for all patients at all times	3.081633	3.074468	
NO22 – B	LSTs should be limited (i.e., not initiated or not augmented) if the patient has a severe neurological injury	2.673469	2.702128	
NO22 – C	LSTs should be withdrawn if the patient has a severe neurological injury	2.806122	2.787234	
*NO22 – D	LSTs should be limited (i.e., not initiated or not augmented) if the patient does not respond to therapy	3.081633	3.106383	Nurses ranked this item lower than physicians.
*NO22 – E	LSTs should be withdrawn if the patient does not respond to therapy	3.040816	3.053191	Nurses ranked this item lower than physicians.
NO22 – F	LSTs should be limited (i.e., not initiated or not augmented) if the patient would not survive the treatment	3.081633	3.074468	
*NO22 – G	LSTs should be withdrawn if the patient would not survive the treatment	2.673469	2.702128	Nurses ranked this item lower than physicians.
NO22 – H	LSTs should be limited (i.e., not initiated or not augmented) if the treatment would only contribute to prolonging the patient’s suffering	2.806122	2.787234	
NO22 – I	LSTs should be withdrawn if treatment only contributes to prolonging the patient’s suffering	4.265306	4.265957	
NO22 – J	LSTs should be limited (i.e., not initiated or not augmented) if the treatment does not ensure the minimum requirements for a dignified life	4	4.042553	
NO22 - K	LSTs should be withdrawn if the treatment does not ensure the minimum requirements for a dignified life	3.989796	4.031915	
NO22 – L	LSTs should be limited (i.e., not initiated or not augmented) if parents ask for LSTs to be stopped	3.55102	3.585106	
NO22 – M	LSTs should be withdrawn if parents ask for LSTs to be stopped	3.489796	3.521277	
**No. 24: Problematic Aspects in Life Supporting Treatment Choices. Based on your experience, what are the most problematic aspects?**				
NO24 – A	Having the responsibility to make the final decision	4.030612	3.989362	
NO24 – B	Not being able to share the decision with others	3.683673	3.680851	
NO24 – C	Lack of clinical ethics consultation	3.5	3.489362	
NO24 – D	The fear of making a wrong choice	3.806122	3.797872	
NO24 – E	Being forced to cause ‘accanimento terapeutico’ (NB: this is an Italian expression referring to persistent needless excessively burdensome interventions, for which there is no directly equivalent term in English) deriving from an orientation of opposition to the withdrawal of LSTs in our PICU	3.877551	3.87234	
NO24 – F	Being forced to cause ‘accanimento terapeutico’ resulting from the opposition of the parents regarding the withdrawal of LSTs in our PICU	3.908163	3.914894	
NO24 – G	Being forced to cause ‘accanimento terapeutico’ for other reasons	3.632653	3.617021	
**No 26: Problematic Aspects in Life Supporting Treatment Choices. Based on your experience, what are the most problematic aspects? (continued)**				
*NO26 – A	Having persistent concerns about possible harms caused to a patient by our actions or decisions	3.704082	3.691489	Nurses ranked this item higher than physicians.
*NO26 – B	Having persistent concerns about possible harms caused to a family by our actions or decisions	3.755102	3.734043	Nurses ranked this item higher than physicians.
NO26 – C	Feeling excluded from the decision-making process	3.459184	3.478723	
NO26 – D	The difficulty in defining solid criteria standards for LST decisions	3.989796	3.989362	
NO26 – E	Having the perception and conviction of using the available (health) resources in an unfair manner	3.642857	3.62766	
NO26 – F	Having the fear of medical-legal consequences resulting from our choices	3.428571	3.425532	
NO26 – G	When my interlocutor (example: parents) has religious convictions that are profoundly different from mine	3.316327	3.329787	
NO26 – H	Fear and fatigue due to the conflict that these choices generate in the team	3.5	3.478723	
NO26 – I	Having no legislative standards for LST decisions	3.938776	3.925532	
NO26 – J	Feeling the need for a legislative framework for end-of-life decision making (example: initiating or withdrawing LSTs)	4.193878	4.180851	
**No 28: SIAARTI: The Italian Society of Anesthesiology, Analgesia, Resuscitation and Intensive Care**; **SARNePI: The Italian society for neonatal and pediatric anesthesia and resuscitation**				
NO28 – A	I know the recommendations on the initiation, continuation and withdrawal of LSTs developed in recent years by SIAARTI and SARNePI	2.816327	2.787234	
NO28 – B	I use the recommendations on the initiation, continuation and withdrawal of LSTs developed in recent years by SIAARTI and SARNePI	2.734694	2.712766	
NO28 – C	In our PICU, it is customary to use the recommendations on the initiation, continuation and withdrawal of LSTs developed in recent years by SIAARTI and SARNePI	2.867347	2.87234	

NB1: All survey items have been translated to English from original Italian survey

• *RN* Nurse

• *MD* Physician

• *LST* life-sustaining treatment

• *PICU*: Pediatric intensive care unit

• Scale 1: Initiate or not initiate (or increase or not increase) LSTs

• Scale 2: Withdraw LSTs

• See Appendix for detailed statistical analyses

(a) whether (i) discussions or responsibility regarding LST decisions involved other physicians within the team (these differences were reported for actual practices as well as for how practices should be) or (ii) if non-initiation of LST is permitted (nurses rated all these items lower than physicians);

(b) (i) LST decision-making criteria are actually based on a patient’s non-survival (referring to LST withdrawal) or prolonged suffering (referring to LST withdrawal and non-initiation) (nurses rated these items higher than physicians); (ii) LST decision-making criteria should be based on a patient’s non-response to treatment (referring to LST withdrawal and non-initiation) or a patient’s non-survival (referring to LST withdrawal) (nurses rated all these items lower than physicians);

(c) concerns about harms caused to patients as well as families because of LST decisions (nurses rated these items higher than physicians).

The second series of analyses examined 27 pairs of questions in the survey for nurses and physicians separately. In this analysis, survey items relating to participants’ ratings of *actual* practices regarding LST decisions were compared with their ratings of how they thought LST decisions *should* be made. These comparisons were analyzed separately for nurses and physicians. For all of these ‘actual/should’ comparisons, comparing 27 pairs of survey questions, statistically significant differences were found for all comparisons among nurses and almost all among physicians, with the exception of three comparisons among the latter highlighted in Table 4. These three comparisons without statistical differences among physicians relate to questions regarding (a) sharing responsibility with parents for non-initiation of LST; (b) non-initiation of LST or (c) LST withdrawal, if parents requested the latter two.

**Table 4:** Comparing participants reports on actual practices with their views on what SHOULD be practiced

Question code	Survey Question (translated from actual survey in Italian)
NO13 – CS1 & NO17 – CS1	The responsibility for the decision is shared with the parents [Scale 1] Responsibility for the decision should be shared with parents [Scale 1]
NO20 – L & NO22 – L	LSTs are not initiated and/or not augmented if parents ask for LSTs to be stopped LSTs should be limited (i.e., not initiated or not augmented) if parents ask for LSTs to be stopped
NO20 – M & NO22 - M	LSTs are withdrawn if parents ask for LSTs to be stopped LSTs should be withdrawn if parents ask for LSTs to be stopped

All comparisons were statistically significant among nurses and among physicians, with the following exceptions

There were no statistically significant differences, among physicians only, for the following paired questions

Questions 24 (items NO24-A to NO24G) and 26 (items NO26-A to NO24-J) asked participants to rate 17 ethical challenges in terms of whether they were problematic. Both nurses and physicians rated all items as problematic, rating all items above 3 and some at 4 or above, on a 5-point scale. Physicians and nurses identified the absence of legislative standards for LST withdrawal as a highly problematic ethical concern. Physicians also identified bearing responsibility for LST decisions as a major concern.

Question 28 included 3 items asking participants to rate their familiarity with and utilization of the recommendations on the initiation, continuation and withdrawal of LSTs developed in recent years by SIAARTI (The Italian Society of Anesthesiology, Analgesia, Resuscitation and Intensive Care) and SARNePI (The Italian society for neonatal and pediatric anesthesia and resuscitation) (2). Nurses and physicians rated all 3 items below 3 on a 5-point scale, demonstrating low levels familiarity and utilization.

Table 5 outlines verbatim exemplars of qualitative data collected in open-ended survey questions, providing additional individual perspectives on some of the survey questions. These data provide more personal accounts of difficult ethical struggles experienced by participants.

**Table 5:** Qualitative Data Analysis

**No. 21: What criteria are used in your ward to make these kinds of LST decisions? [Please specify]** • It depends on how and by whom the situation is explained, often a minimum of hope is promoted even when it is not there (RN) • If the health care team shares the parents’ choice (MD) • If the parents’ decision is not similar to that of the health care team (MD)	
**No 23: In your opinion, what criteria do you think should be used? [Please specify]** • I believe that the decision to limit or suspend life support should ALWAYS be made collectively by parents, nurses and doctors, and that doctors should give accurate information to parents to enable them to make informed decisions (RN) • I think it is not up to us to judge what is dignified or not, we are no one to decide that a person ‘must’ die, we are no one even to say that they ‘must’ live (RN) • We should always have the intellectual honesty to communicate the real situation and be able to share with the whole team and parents (even the patient if we are dealing with a teenager) and evaluate case by case the best treatment and solutions (RN) • Giving false hopes or harassing defenseless people is cowardly and disrespectful (RN) • Seek to share decisions with parents (MD)	
**No. 25: Problematic aspects of LST decisions. In your experience; what are the most problematic aspects? [Please specify]** • In general, the main problem lies not in the parents who best of all understand the suffering of the child but in the orientation contrary to the withdrawal of care that denotes the culture of doctors, in particular of the senior physician responsible for the PICU, who never wants to involve the Clinical Ethics Committee in any way and leaves the whole burden of decisions and interviews with parents to the doctor on duty, generally young physicians on night duty. After a death, none of the doctors ever want to talk about the case again. Moreover, even some young doctors, just to avoid problems, are willing to sustain ‘accanimento terapeutico’. The nursing staff, on the other hand, is always more sensitive and available for meetings to discuss such cases (MD) • Caused by not feeling protected (RN) • The opinions of members of the treating team cannot always be aligned. In these circumstances the opinion of the ethics committee is useful in orienting and choosing a common line, even if not always fully shared by everyone. Sometimes a strong parental opinion can force the team to maintain or continue care that is futile or does not ensure a minimum quality of life for the child (MD) • Unfortunately, in our reality the withdrawal of some vital supports is not always accepted by everyone and therefore sometimes a limitation of treatments is decided (rather than withdrawal) (MD) • Different theories and ideologies of the various doctors on the team (RN) • Often we are afraid of the consequences and prejudices of people, the law often does not even protect professionals. The choice of ‘accanimento terapeutico’ is therefore understandable at times but only for personal protection. With the ‘living will’ something could change for adults, but for pediatrics I am not optimistic (RN) • ‘accanimento terapeutico’ is used as defensive medicine (RN)	
**No 27: Other: Please specify (if forced to cause ‘accanimento terapeutico’ for other reasons)** • The massive waste of economic resources is really a HUGE problem in my opinion. It’s a question I ask myself every day! (MD) • The absence of a CLEAR legislative framework also gives way to a thousand interpretations and above all does not indicate a common approach. The lack of a true ethics consultation (the American model for example) is a serious problem. The [name of hospital is anonymized] Ethics Committee is composed of random people with no experience in resuscitation, and the only intensivist involved is not in the least taken into consideration by the top intensivists who are definitively pro-‘accanimento terapeutico’. The problem is serious and it is the principal cause of burnout among medical and nursing staff (MD)	
**No: 29: Other Comments:** • Every single case deserves a collegial discussion. In emergency situations, we often find ourselves in the position of having to start life support, even invasive interventions. It is not always easy then when the case becomes oriented toward a poor prognosis and the withdrawal of LST should be undertaken (MD) • I am a simple nurse and in the face of life events, where we have to decide, I find myself in difficulty regarding the certainty dictated by people superior to me. I believe that in suffering there is no man capable of deciding whether he is right or not, whether he is a head physician or a nurse. Faced with a life touched by a profound problem, where rationality leads us to decide, I listen and let myself be carried away by Faith that helps me to live linked to principles that are important to me (RN) • Greater support on a psychological and emotional level for staff and parents in the post-mortem and better decision-making would be useful (RN) • Many circumstances are interfered with by ‘team’ orders and by the fear of those responsible for running into medico-legal situations that could expose them to criticism and denunciations (MD) • What is missing, in addition to the advice of an ethicist which fortunately would be requested only a few times a year, is NEEDED PSYCHOLOGICAL SUPPORT for the critical care team which would serve to consolidate and amalgamate complex decisions by analyzing the positions of individual members and possibly solving impasses with individuals who have a conflicting view given their subjective experience with end-of-life problems (MD) • How to establish the concept of “a dignified life” in a manner that is valid for the whole team (RN)	

NB: Excerpts of all qualitative data are presented, to demonstrate a range of views disclosed by nurses and physicians

NB: All survey questions and replies have been translated to English from original Italian survey

• ‘Accanimento terapeutico’: This is an Italian expression referring to persistent needless excessively burdensome interventions, for which there is no directly equivalent term in English.

• *LST* Life-sustaining treatment

• *RN* Nurse

*MD* Physician.

## Discussion

Data generated by this investigation have corroborated international research results regarding the many significant ethical challenges confronted by PICU HCPs, as well as our own previous qualitative research within Italy. The magnitude of these ethical challenges among participants in this study was revealed through a particular feature of our study design. Comparatively analyzing participants’ reports of actual LST decision-making practices contrasted with their views on how these decisions should be made – directly comparing *actual* with *should* – helped bring to light the many facets of current practices that participants considered ethically inadequate. Indeed, ethical tensions were identified across all the realms of LST decision-making practices that were examined. Results drawn from these multiple Italian sites as well as inter-professional participants (i.e., nurses and physicians) suggest that PICU teams are commonly confronting significant ethical difficulties that are inadequately addressed. Our results also demonstrated that some ethical challenges are experienced differently according to professional perspectives - i.e., nursing or medicine – corroborating our earlier qualitative research which revealed the many differences in roles, responsibilities, and ethical difficulties encountered within these two professions [8]. Quantitative results were further illuminated by qualitative data.

These results highlight needed substantive and procedural advances regarding ethical aspects of PICU practice (e.g., policies, practice standards). Substantively, following from results reported in our previous study, PICU HCPs are troubled by the lack of clear legal or ethical standards regarding the permissibility of withdrawing LST for children. This is especially noteworthy when compared to some other countries where there are no legal or ethical distinctions between non-initiation and withdrawal of LST, basing such decisions on a case-by-case basis in terms of the best interests of the child in question [5, 6]. Moreover, substantive standards could also clarify the formal role and responsibility that parents should have regarding LST decisions in the PICU. Some LST decision-making standards already existed at the time of the study, such as the SIAARTI guidelines [2]. Such standards were developed to serve as professional practice supports, without explicit grounding in Italian legal norms. Yet, participants demonstrated a low level of awareness of these standards. Recognized national professional societies could lead the further development of such standards and the promotion of their eventual legal recognition by legislators. Indeed, although the recent Italian LAW n. 219, 22 December 2017 - operational since January 16, 2018 - established new rules on informed consent and advance care planning, these have not explicitly defined LST decision standards for pediatrics.

In terms of needed procedural advancements, some differences were noted between nurses’ and physicians’ accounts of how LST decisions were made, while some participants reported that psychological and clinical ethics supports are needed to assist HCPs and parents to navigate these complex decisions that involve numerous stakeholders. Procedural advancements could include the development of practice standards and institutional policies that promote the involvement of consultants with psychological and clinical ethics expertise to support PICU teams and families to help ensure open discussions, collaborative and respectful communication, constructive reconciliation of disagreements, individual and group opportunities to address experiences with moral distress, and treatment decision-making aligned with relevant national and international legal, ethical, and professional standards [15]. This can include the development of policies on the use of consultations with a clinical ethics committee or consultant for cases that are actually or likely to be ethically troublesome.

Educational activities should be organized within hospital centers and within regional and national conferences to help PICU HCPs learn about substantive and procedural strategies for addressing ethical concerns within their practice.

Future research should extend the investigation reported here in additional PICUs throughout Italy. Such research should solicit participation from PICUs in southern Italy, where ethical views and practices regarding LSTs may differ from those in northern and central Italy. We acknowledge the non-participation of southern Italian PICUs as a limitation of the study. Moreover, future research should investigate LST decisions over time, examining the course of *actual* clinical practices in relation to what clinicians think *should* be done.

Another limitation of this study was that the sample sizes required for t-test analyses could not be attained within a reasonable timeframe. On the other hand, sound data analyses could still be conducted with nonparametric robust statistical methods [9].

## Conclusion

The results of this study highlight a need for the development of (a) strategies for improving team processes regarding LST decisions, so they can be better aligned with how clinicians think decisions should be made, and (b) Italian LST decision-making standards that can help ensure optimal ethical practices.

## Data Availability

The datasets used for this study are available from the corresponding author on reasonable request.

## Appendix

### Detailed Statistical Analyses

NB: All robust tests were conducted using the `WRS' or the `WRS2' packages in R version 3.6. For each series of comparisons, a sample R code for the rst comparison is provided.

### Primary Research Question (RQ1)

This series of analyses were conducted to compare doctors' vs. nurses' responses for all 88 questions in the survey. For this set of independent mean comparisons, Yuen's (1974) modied t-test for independent trimmed means with 5000 bootstrap was used (Field & Wilcox, 2017; Wilcox & Rousselet, 2018). In order to adjust for multiple comparisons, Benjamin-Hochberg procedure was used (Benjamini & Hochberg, 1995; McDonald, 2014) whereas the original *p*-values were reported in the table.

Notes

- Nurse = 1; Doctor = 2
- Sample R code: yuenbt(No12AS1~Role,data=PFData,nboot=5000)
- Trimmed mean at 20%
- indicates statistical significance after multiple testing correction using Benjamin-Hochberg procedure

**Table:**

Comparisons	Mdiff	95%CI	Yt	***p*** -value
No12AS1	0.27	[-0.35, 0.88]	0.96	.36
No12AS2	-0.13	[-1.12, 0.86]	-0.33	.75
No12BS1	-0.48	[-0.89, -0.06]	-2.23	.02
No12BS2	-0.61	[-0.97, -0.25]	-3.60	.003*
No12CS1	-0.48	[-1.12, 0.17]	-1.48	.14
No12CS2	-0.53	[-1.31, 0.25]	-1.37	.17
No13AS1	-0.05	[-0.70, 0.60]	-0.15	.88
No13AS2	0.19	[-0.38, 0.76]	0.65	.52
No13BS1	-0.37	[-0.71, -0.03]	-2.11	.04
No13BS2	-0.61	[-0.95, -0.26]	-3.50	.008*
No13CS1	0.29	[-0.05, 0.64]	1.64	.09
No13CS2	0.40	[-0.15, 0.95]	1.50	.15
No13DS1	-0.27	[-0.96, 0.42]	-0.81	.40
No13DS2	-0.35	[-0.98, 0.28]	-1.10	.27
No14A	-0.92	[-1.35, -0.49]	-4.15	.0008*
No14B	-0.38	[-0.98, 0.22]	-1.30	.21
No14C	0.15	[-0.48, 0.79]	0.54	.58
No14D	-0.10	[-0.62, 0.43]	-0.39	.70
No14E	0.31	[-0.50, 1.13]	0.81	.42
No15A	0.41	[-0.42, 1.23]	1.04	.32
No15B	0.02	[-0.60, 0.64]	0.07	.94
No16BS1	-0.37	[-0.61, -0.13]	-3.11	0.006*
No16BS2	-0.36	[-0.56, -0.16]	-3.50	.001*
No16AS1	-0.11	[-0.47, 0.24]	-0.63	.52
No16AS2	-0.22	[-0.58, 0.13]	-1.21	.22
No16CS1	-0.06	[-0.42, 0.29]	-0.34	.72
No16CS2	-0.14	[-0.51, 0.22]	-0.80	.43
No17AS1	0.02	[-0.39, 0.43]	0.12	.94
No17AS2	0.23	[0.03, 0.43]	2.34	.03
No17BS1	-0.30	[-0.67, 0.06]	-1.81	.08
No17BS2	-0.38	[-0.59, -0.18]	-3.73	.0006*
No17CS1	0.41	[-0.41, 1.23]	1.11	.29
No17CS2	0.38	[-0.50, 1.27]	1.00	.34
No17DS1	0.01	[-0.34, 0.37]	0.08	.94
No17DS2	-0.14	[-0.50, 0.21]	-0.79	.42
No18A	-0.48	[-0.92, -0.04]	-2.20	.03
No18B	-0.30	[-0.70, 0.10]	-1.71	.10
No18C	0.06	[-0.29, 0.41]	0.34	.71
No18D	-0.12	[-0.48, 0.24]	-0.68	.50
No18E	0.23	[-0.38, 0.84]	0.86	.38
No19A	0.06	[-0.29, 0.41]	0.35	.74
No19B	0.06	[-0.28, 0.40]	0.34	.72
No20A	0.48	[-0.32, 1.28]	1.25	.22
No20B	-0.30	[-1.01, 0.40]	-0.93	.35
No20C	-0.47	[-1.07, 0.13]	-1.60	.11
No20D	-0.33	[-0.84, 0.18]	-1.34	.20
No20E	-0.33	[-0.91, 0.25]	-1.26	.21
No20F	-0.39	[-0.99, 0.21]	-1.33	.19
No20G	-0.52	[-0.94, -0.11]	-2.42	.01*
No20H	-0.92	[-1.50, -0.33]	-3.16	.003*
No20i	-0.76	[-1.38, -0.14]	-2.51	.02*
No20J	-0.67	[-1.26, -0.08]	-2.45	.03
No20K	-0.07	[-0.63, 0.48]	-0.28	.78
No20L	0.13	[-0.49, 0.75]	0.44	.67
No20M	0.15	[-0.44, 0.75]	0.55	.57
No22A	0.61	[-0.64, 1.86]	1.16	.27
No22B	-0.14	[-0.75, 0.47]	-0.46	.63
No22C	-0.32	[-0.90, 0.27]	-1.08	.29
No22D	-0.70	[-1.22, -0.19]	-2.68	.01*
No22E	-0.78	[-1.30, -0.26]	-2.97	.006*
No22F	-0.42	[-0.84, -0.006]	-1.95	.05
No22G	-0.66	[-1.08, -0.23]	-3.09	.007*
No22H	-0.35	[-0.71, 0.004]	-2.01	.06
No22i	-0.35	[-0.70, -0.01]	-2.05	.05
No22J	-0.16	[-0.78, 0.45]	-0.55	.59
No22K	-0.29	[-0.93, 0.35]	-0.97	.34
No22L	0.14	[-0.67, 0.96]	0.37	.72
No22M	0.04	[-0.86, 0.94]	0.10	.92
No24A	0.62	[-0.36, 1.60]	1.67	.15
No24B	-0.55	[-1.15, 0.06]	-1.86	.07
No24C	0.35	[-0.26, 0.96]	1.24	.23
No24D	0	[-0.82, 0.82]	0	1
No24E	-0.26	[-0.86, 0.34]	-0.90	.36
No24F	-0.47	[-0.89, -0.06]	-2.21	.03
No24G	-0.33	[-0.90, 0.23]	-1.21	.23
No26A	-0.65	[-1.00, -0.29]	-3.61	.001*
No26B	-0.65	[-1.00, -0.29]	-3.61	.001*
No26C	0.22	[-0.40, 0.84]	0.78	.44
No26D	-0.29	[-0.85, 0.26]	-1.04	.30
No26E	-0.54	[-1.19, 0.10]	-1.81	.09
No26F	0.33	[-0.40, 1.05]	0.94	.35
No26G	0.56	[-0.002, 1.23]	1.96	.05
No26H	0.04	[-0.62, 0.70]	0.13	.90
No26i	0.05	[-0.57, 0.67]	0.17	.87
No26J	0.12	[-0.40, 0.64]	0.46	.64
No28A	-0.49	[-1.07, 0.09]	-1.76	.09
No28B	-0.18	[-0.77, 0.41]	-0.67	.50
No28C	0.40	[-0.02, 0.81]	1.89	.06

### Second Research Question (RQ2)

This series of analyses were conducted to examine 27 pairs of questions in the survey for nurses and doctors separately. For this set of dependent mean comparisons, procedure using 20% trimmed mean with 5000 percentile bootstrap was used (Wilcox, 2017).

Notes

- Sample R code: dtrimpb(N1, alpha=0.05, con=0, est=tmean, plotit=FALSE, nboot=5000)
- Trimmed mean at 20% • No multiple testing correction was performed
- † indicates statistical significance at the 95% CI

**Table:**

	Nurse		Doctor	
Comparisons	Ψ	95%CI	Ψ	95%CI
No12AS1 & No16AS1	-0.49†	[-0.72, -0.28]	-0.58†	[-1.11, -0.21]
No12BS1 & No16BS1	-0.33†	[-0.64, -0.13]	-0.16†	[-0.58, 0]
No12CS1 & No16CS1	-1.28†	[-1.77, -0.79]	-0.79†	[-1.47, -0.32]
No13AS1 & No17AS1	0.44†	[0.23, 0.67]	0.42†	[0.16, 0.89]
No13BS1 & No17BS1	-0.28†	[-0.49, -0.10]	-0.29†	[-0.81, -0.05]
No13CS1 & No17CS1	-0.36†	[-0.59, -0.15]	-0.26	[-0.68, 0.16]
No13DS1 & No17DS1	-1.10†	[-1.62, -0.64]	-0.79†	[-1.26, -0.42]
No14A & No18A	-1.00†	[-1.46, -0.69]	-0.63†	[-0.95, -0.37]
No14B & No18B	-0.77†	[-1.15, -0.46]	-0.58†	[-1.11, -0.21]
No14C & No18C	-0.36†	[-0.59, -0.15]	-0.37	[-0.74, 0]
No14D & No18D	-0.56†	[-0.87, -0.36]	-0.53†	[-0.84, -0.21]
No14E & No18E	-0.92†	[-1.36, -0.56]	-1.00†	[-1.58, -0.58]
No15A & No19A	-0.59†	[-0.92, -0.33]	-0.95†	[-1.63, -0.37]
No15B & No19B	-1.13†	[-1.67, -0.62]	-1.11†	[-1.74, -0.58]
No20A & No22A	0.74†	[0.38, 1.10]	0.84†	[0.32, 1.47]
No20B & No22B	-1.36†	[-1.77, -1.00]	-1.05†	[-1.68, -0.47]
No20C & No22C	-0.97†	[-1.41, -0.62]	-0.89†	[-1.53, -0.32]
No20D & No22D	-1.18†	[-1.41, -0.90]	-1.05†	[-1.63, -0.58]
No20E & No22E	-0.95†	[-1.26, -0.64]	-0.95†	[-1.53, -0.47]
No20F & No22F	-1.15†	[-1.44, -0.82]	-0.79†	[-1.32, -0.26]
No20G & No22G	-0.97†	[-1.28, -0.67]	-0.84†	[-1.32, -0.42]
No20H & No22H	-1.21†	[-1.69, -0.79]	-0.53†	[-1.05, -0.26]
No20I & No22I	-1.18†	[-1.64, -0.82]	-0.84†	[-1.37, -0.42]
No20J & No22J	-1.49†	[-1.85, -1.13]	-0.74†	[-1.37, -0.32]
No20K & No22K	-1.10†	[-1.46, -0.77]	-1.05†	[-1.74, -0.53]
No20L & No22L	-0.41†	[-0.74, -0.21]	-0.32	[-0.63, 0.11]
No20M & No22M	-0.41†	[-0.72, -0.21]	-0.37	[-0.68, 0]
